# Clinical diagnostic evaluation of HRP2 and pLDH-based rapid diagnostic tests for malaria in an area receiving seasonal malaria chemoprevention in Niger

**DOI:** 10.1186/s12936-019-3079-1

**Published:** 2019-12-26

**Authors:** Matthew E. Coldiron, Bachir Assao, Céline Langendorf, Nathan Sayinzoga-Makombe, Iza Ciglenecki, Roberto de la Tour, Erwan Piriou, Mahaman Yarima Bako, Ann Mumina, Ousmane Guindo, Anne-Laure Page, Rebecca F. Grais

**Affiliations:** 1 Epicentre, 8 rue Saint-Sabin, Paris, France; 2 Epicentre, Maradi, Niger; 3 Médecins Sans Frontières, 70 rue de Lausanne, Geneva, Switzerland; 4 Médecins Sans Frontières, Naritaweg 10, Amsterdam, The Netherlands; 5 Ministry of Public Health, Magaria, Niger; 6 Médecins Sans Frontières, Niamey, Niger

**Keywords:** Malaria, Rapid diagnostic test, *Plasmodium* lactate dehydrogenase, Histidine-rich protein 2, Niger

## Abstract

**Background:**

Rapid diagnostic tests (RDT) for malaria are common, but their performance varies. Tests using histidine-rich protein 2 (HRP2) antigen are most common, and many have high sensitivity. HRP2 tests can remain positive for weeks after treatment, limiting their specificity and usefulness in high-transmission settings. Tests using *Plasmodium* lactate dehydrogenase (pLDH) have been less widely used but have higher specificity, mostly due to a much shorter time to become negative.

**Methods:**

A prospective, health centre-based, diagnostic evaluation of two malaria RDTs was performed in rural Niger during the high malaria transmission season (3–28 October, 2017) and during the low transmission season (28 January–31 March, 2018). All children under 5 years of age presenting with fever (axillary temperature > 37.5 °C) or history of fever in the previous 24 h were eligible. Capillary blood was collected by finger prick. The SD Bioline HRP2 (catalog: 05FK50) and the CareStart pLDH(pan) (catalog: RMNM-02571) were performed in parallel, and thick and thin smears were prepared. Microscopy was performed at Epicentre, Maradi, Niger, with external quality control. The target sample size was 279 children with microscopy-confirmed malaria during each transmission season.

**Results:**

In the high season, the sensitivity of both tests was estimated at > 99%, but the specificity of both tests was lower: 58.0% (95% CI 52.1–63.8) for the pLDH test and 57.4% (95% CI 51.5–63.1) for the HRP2 test. The positive predictive value was 66.3% (95% CI 61.1–71.2) for both tests. In the low season, the sensitivity of both tests dropped: 91.0% (95% CI 85.3–95.0) for the pLDH test and 85.8% (95% CI 79.3–90.9) for the HRP2 test. The positive predictive value remained low for both tests in the low season: 60.5% (95% CI 53.9–66.8) for the pLDH test and 61.9% (55.0–68.4) for the HRP2 test. Performance was similar across different production lots, gender, age of the children, and, during the high season, time since the most recent distribution of seasonal malaria chemoprevention.

**Conclusions:**

The low specificity of the pLDH RDT in this setting was unexpected and is not easily explained. As the pLDH test continues to be introduced into new settings, the questions raised by this study will need to be addressed.

## Background

Rapid diagnostic tests (RDT) for malaria have become the in-the-field diagnostic standard in most countries in Africa. Current malaria RDTs are immunochromatographic tests that detect the presence of circulating parasite antigens. The two most commonly targeted antigens are histidine-rich protein 2 (HRP2), which is specific to *Plasmodium falciparum,* and *Plasmodium* lactate dehydrogenase (pLDH), which is present in all *Plasmodium* species; currently available tests detect one or both antigens. HRP2-based tests have been preferred in areas where *P. falciparum* is predominant, due to a higher reported sensitivity [1]. There is also some evidence that they are more heat-stable than pLDH-based tests [2]. In vivo, the two antigens are cleared with different speeds, which appears to affect their specificities (notably, their ability to detect current infection) depending on the context. An evaluation in both high- and low-transmission areas in Uganda showed that median time for an HRP2 test to become negative after an effective treatment was 35–42 days, but that the median time to become negative for a pLDH test was only 2 days [3]. This likely explained the higher specificity of the pLDH test (94%) compared to HRP2 test (80%) in the high-transmission area, while specificities were similarly high (~ 99%) in the low-transmission area. The higher specificity of pLDH tests was also seen in hospitalized children in Burkina Faso [4]. These findings have important implications in high-transmission environments, where interpretation of HRP2 tests can be difficult in the peak malaria season, leading to false positive results and hence unnecessary treatments. In part because of these results, a pLDH-based malaria RDT was introduced by the medical humanitarian organization Médecins Sans Frontières in several health structures it supports in the Magaria District of Niger. In all other health centres of the District, and throughout the rest of Niger, HRP2-based tests are used by the Ministry of Public Health and its partners.

The CareStart™ Malaria PAN (pLDH) Ag RDT (Reference RMNM-02571, hereafter referred to as CareStart pLDH(pan) is among the highest-scoring RDTs in the most recent WHO-sponsored evaluation of RDTs [5]. These evaluations do not formally estimate the sensitivity and specificity of the RDTs, but instead use a geographically diverse set of blood samples with known levels of parasitaemia to calculate a ‘Panel Detection Score’ (PDS). In the most recent round, the SD BIOLINE Malaria Antigen (Reference 05FK50, hereafter referred to as SD Bioline HRP2) test had a PDS > 90% for the lowest level parasitaemia (200 parasites/µl), and a false positivity rate < 2%, both of which comfortably meet WHO thresholds for performance. At the lowest level parasitaemia, the PDS for the Carestart pLDH(pan) was slightly lower at 84–86%, but still comfortably met the thresholds, and the false-positivity rate was 0. However, the testing is performed in ideal conditions, so the conclusions are not always transferrable to field conditions. In the recent study in Uganda, sensitivity of the Carestart pLDH (pan) test was estimated at 96.1% (95% CI 92.9–98.1) and 94.7% (95% CI 91.2–97.0) in areas of low and high malaria transmission, respectively; its specificity was estimated at 99.8% (99.6–99.9) and 93.9% (89.6–96.8), respectively [3]. In the same study, the positive predictive values of the test were above 95% in both high and low-transmission environments.

A formal clinical diagnostic evaluation of the newly introduced pLDH-based RDT, in parallel with the previously used HRP2-based RDT, was performed in field conditions in rural Niger. Off-site microscopy by at least two blinded microscopists was the gold standard comparator.

## Methods

### Study design and setting

Malaria transmission in Niger has a marked seasonality, with a large peak between July and October, that follows the rainy season (June–September). As of 23 December, a total of 3,484,069 cases of malaria were reported across the country in 2018, including 106,004 cases in Magaria District [6]. Magaria District is in the target zone for seasonal malaria chemoprevention (SMC), a strategy which administers monthly courses of sulfadoxine-pyrimethamine and amodiaquine to children aged 3–59 months during the rainy season, and which has been recommended by the WHO since 2012 [7].

**Fig. 1:**
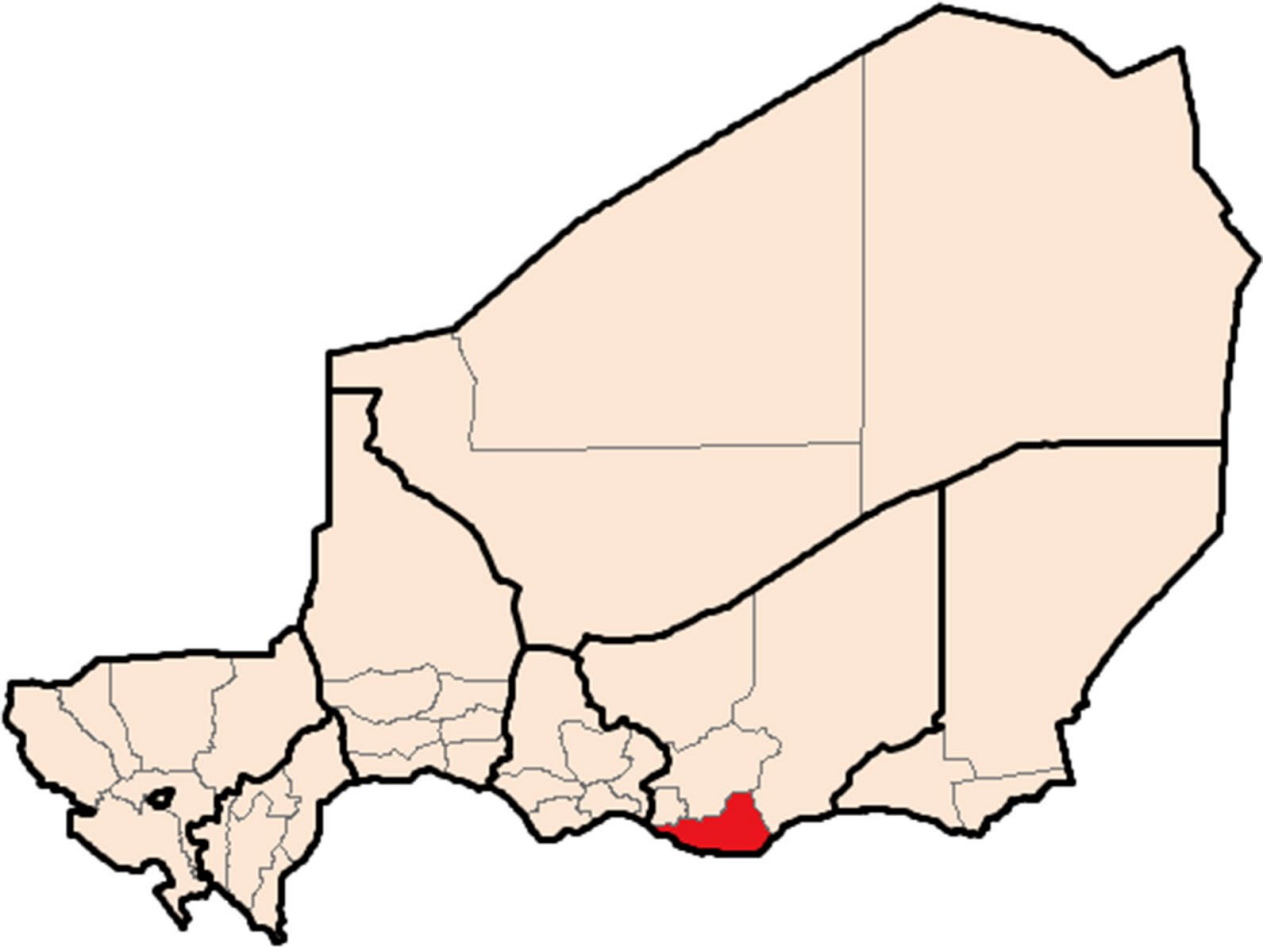
Magaria District in the Zinder Region of Niger

A prospective, health centre-based, clinical diagnostic evaluation was performed in two phases: first during the peak malaria transmission season (3–28 October, 2017) and then during the low malaria transmission season (28 January–31 March, 2018). In October 2017, the timing ensured that inclusions happened over the course of an entire monthly cycle of SMC, which has been offered in the study area since 2013. In the high season, the study took place in the Magaria Integrated Health Centre (Centre de Santé Intégré (CSI)), an outpatient facility in Magaria, Niger (Fig. 1). During the low season, the study began in the Magaria CSI, but due to low numbers of inclusions, a second study site was added at the Dantchiao CSI, approximately 30 km to the east of the Magaria CSI.

### Sample collection and processing

Children aged 3–59 months (the target age group of SMC) presenting to study sites with fever (37.5 °C axillary temperature) or a history of fever in the preceding 24 h were enrolled. Children with any sign of severity (including but not limited to decreased consciousness and seizure) were excluded and immediately referred to the District Hospital for treatment. During the high transmission season, in order to spread inclusions throughout the period between SMC distributions, only the first 20 febrile children presenting each day were enrolled. During the low transmission season, all febrile children presenting at the study CSI were offered enrolment.

After obtaining written informed consent from a participant’s caregiver, a study nurse collected demographic and clinical information about the participant, including age, gender, village of origin, bed-net use, receipt of most recent SMC (high season only), and information about the RDT used (lot number, date/time of performance of test). The study nurse collected capillary blood by a fingerstick, preparing thick and thin smears in duplicate. The study nurse wrote the patient ID number and time of testing on the RDT cassettes. The two tests (SD Bioline Ag P.f. (HRP2), Standard Diagnostics, Giheung-ku, Republic of Korea, catalog number 05FK50; and CareStart pLDH(pan), AccessBio, Somerset, NJ, USA, catalog number RMNM-02571) were performed following manufacturers’ instructions. A photograph of the RDT was taken at the time of reading (15 min for the HRP2 test and 20 min for the pLDH test), and the results were recorded in the study register. Invalid RDTs were repeated for clinical purposes but only the first result was considered for the diagnostic evaluation.

Any child with a positive result on either test (pLDH or HRP2) was treated, free of charge, following national protocols, with antipyretics, artemether–lumefantrine, and any other treatment deemed necessary by the treating clinician. Febrile children with a negative RDT were treated according to the clinician’s best judgment, and all treatment was also free of charge.

### Laboratory methods

At the end of each day, the slide of each participant that appeared to be of the best quality was stained (10% Giemsa solution for 15 min) and the lesser-quality slide was discarded. Slides were stored and regularly transported to the Epicentre Laboratory in Maradi, where microscopy was carried out according to WHO standards by a team of expert microscopists with over 20 years combined experience in malaria microscopy [8]. In brief, the microscopists performed blind double-reading of all slides, with 200 high-power fields for negativity, and a third reading by a blinded microscopist in case of discrepant results between the first two for detection, species identification or parasitaemia discrepancy > 50%. For positive slides, results included parasitaemia (sexual forms only) and species identification. The presence or absence of gametocytes was noted for all samples. Ten percent of negative and 10% of positive slides were sent for external quality control at the Centre de Recherche Médicale et Sanitaire, in Niamey.

Following manufacturer’s instructions, the RDTs were stored at temperatures < 30 °C between shipment and their arrival to the study site. At the study sites, RDTs were stored at ambient temperature in a ventilated, locked room for the duration of the data collection period (4 weeks during the high transmission season; 8 weeks during low transmission season). At least two different production lots were used during each transmission season.

### Statistical methods and sample size

The target sample size was calculated based on the number of true-positive cases needed to ensure that sensitivity and specificity (lower bound of the 95% CI) are above 90%, with 90% power and alpha = 5%, under the null hypothesis that the sensitivity and specificity are 95% and 98%, respectively. Under this scenario, a target of 279 children with microscopy-positive malaria during each transmission season was set. In the high transmission season, when, based on previous observational data from health clinics in the area, the assumed prevalence of microscopy positivity was 60% among febrile children, the target sample size for enrolment was therefore 465 febrile children. In the low-transmission season, assuming the prevalence of microscopy positivity would be 30% among febrile children, the original target sample size was 930 children. Under those assumptions, this would provide enough microscopy-negative samples to meet the desired precision. During the low season, it became obvious that the true prevalence was lower than 30%, and that enrolling only 930 participants would not give the 279 true positives necessary. (The rate of RDT positivity was only 10% after the first week). Inclusions were therefore extended through 31 March, 2018, the longest period logistically possible to carry out the study, and a second study site was also added at the Dantchiao CSI.

The performance characteristics of the RDTs (sensitivity, specificity, positive predictive value (PPV), negative predictive value (NPV)) were calculated independently during each transmission period, and 95% CIs were calculated using the exact binomial method. Only infections with *P. falciparum* (mono-infection or co-infection) were considered positive for analytic purposes. Comparisons in sub-groups should be regarded as exploratory, as the target sample size was not set with the goal of making these comparisons. Data were analysed using Stata version 15 (College Station, TX, USA).

## Results

### Description of participants

A total of 539 participants were enrolled in the high transmission season, including 246 (46%) with *P. falciparum* parasitaemia by microscopy, and 1407 participants were enrolled in the low transmission season, including 155 (11%) with *P. falciparum* parasitaemia by microscopy (Table 1). During the high transmission season, 517 participants (96%) reported sleeping under a bed net the night prior to enrolment, and 354 (66%) reported having received the most recent distribution of SMC. During the low transmission season, 862 (61%) participants reported sleeping under a bed net the night prior to enrolment. Full results of microscopy are presented in Table 2. During the high transmission season, 3 cases had mixed *P. falciparum*/*Plasmodium ovale* infection and 2 had *P. ovale* mono-infection. During the low season, in addition to the 155 participants with *P. falciparum* parasitaemia, 4 participants had *Plasmodium malariae* parasitaemia.

**Table 1:** Demographic characteristics of participants and description of malaria parasitaemia, Magaria, Niger, 2017–2018

	High transmission season (N = 539)	Low transmission season (N = 1407)
Gender, n (%)		
M	292 (54)	760 (54)
F	247 (46)	647 (46)
Age in months		
3–11	148 (28)	557 (40)
12–23	166 (31)	526 (37)
24–35	129 (24)	205 (14)
36–47	51 (19)	67 (5)
48–59	45 (8)	52 (4)
Median age in months (IQR)	18 (10–24)	12 (8–22)
*P. falciparum* parasitaemia n (%)	246 (46)	155 (11)
Median parasitaemia (IQR), asexual parasites/µl	12,684 (2682–50,958)	624 (119–3010)
< 200/µl, n (%)	11 (4)	54 (35)
200–1999/µl, n (%)	42 (17)	54 (35)
2000–199,999/µl, n (%)	179 (73)	46 (30)
≥ 200,000/µl, n (%)	14 (6)	1 (1)
*P. falciparum* gametocytaemia, n (%)	95 (18)	79 (6)

**Table 2:** Description of parasitaemia by age, Magaria, Niger 2017–2018

Age	High transmission season	Low transmission season
3–11 months		
*P. falciparum* parasitaemia, n/N (%)	45/148 (30)	34/557 (6)
Median parasitaemia (IQR), asexual parasites/µl	5851 (1209–31,423)	490 (119–1576)
Parasite density, asexual parasites/µl, n/N (%)		
< 200	1/45 (2)	13/34 (38)
200–1999	14/45 (31)	14/34 (41)
2000–199,999	28/45 (62)	7/34 (21)
≥ 200,000	2/45 (4)	0/34 (0)
Gametocytaemia, n/N (%)	21/148 (14)	21/557 (4)
12–23 months		
*P. falciparum* parasitaemia, n/N (%)	58/166 (35)	53/526 (10)
Median parasitaemia (IQR), asexual parasites/µl	12,092 (2273–48,385)	444 (71–1983)
Parasite density, asexual parasites/µl, n/N (%)		
< 200	3/58 (5)	22/53 (42)
200–1999	9/58 (16)	19/53 (36)
2000–199,999	45/58 (76)	12/53 (23)
≥ 200,000	2/58 (3)	0/53 (0)
Gametocytaemia, n/N (%)	27/166 (16)	27/526 (5)
24–35 months		
*P. falciparum* parasitaemia, n/N (%)	79/129 (61)	39/205 (19)
Median parasitaemia (IQR), asexual parasites/µl	16,858 (4039–66,324)	2675 (197–18,673)
Parasite density, asexual parasites/µl, n/N (%)		
< 200	6/79 (8)	10/39 (26)
200–1999	9/79 (11)	9/39 (23)
2000–199,999	59/79 (75)	19/39 (49)
≥ 200,000	5/79 (6)	1/39 (3)
Gametocytaemia, n/N (%)	33/129 (26)	19/205 (9)
36–47 months		
*P. falciparum* parasitaemia, n/N (%)	32/51 (63)	15/67 (22)
Median parasitaemia (IQR), asexual parasites/µl	46,571 (5571–113,900)	1198 (190–2732)
Parasite density, asexual parasites/µl, n/N (%)		
< 200	0/32 (0)	4/15 (27)
200–1999	5/32 (16)	5/15 (33)
2000–199,999	24/32 (75)	6/15 (40)
≥ 200,000	3/32 (9)	0/15 (0)
Gametocytaemia, n/N (%)	7/51 (14)	9/67 (13)
48–59 months		
*P. falciparum* parasitaemia, n/N (%)	32/45 (71)	14/52 (27)
Median parasitaemia (IQR), asexual parasites/µl	14,886 (2542–46,578)	383 (167–1012)
Parasite density, asexual parasites/µl, n/N (%)		
< 200	1/32 (3)	5/14 (36)
200–1999	5/32 (16)	7/15 (50)
2000–199,999	24/32 (75)	2/50 (14)
≥ 200,000	2/32 (6)	0/14 (0)
Gametocytaemia, n/N (%)	7/45 (16)	3/52 (6)

### Performance characteristics of RDTs

In the high season, for the pLDH test, there were 361 positive results, 169 negative results, and 9 invalid results; for the HRP2 test, there were 370 positive results and 169 negative results. In the low season, for the pLDH test, there were 233 positive results, 1173 negative results and 1 invalid result; for the HRP2 test, there were 215 positive results and 1192 negative results.

In the high season, the sensitivity of both tests was estimated at > 99%, but the specificity of both tests was around 58% (Table 3). The point estimate of the PPV for both tests was 66.3%. In the low season, the sensitivity of both tests was lower: 91.0% for the pLDH test and 85.8% for the HRP2 test. And although the specificity of both tests was higher, given the much lower disease prevalence, the PPV of a positive result remained low for both tests: 60.5% for the pLDH test and 61.9% for the HRP2 test.

**Table 3:** Performance characteristics of two malaria rapid diagnostic tests, Magaria, Niger, 2017–2018

Characteristic	High transmission season			Low transmission season		
Test	N^a^	Value	95% CI	N^a^	Value	95% CI
Sensitivity						
pLDH	246	99.2	97.0–99.9	155	91.0	85.3–95.0
HRP2	246	99.2	97.1–99.9	155	85.8^‡^	79.3–90.9
Specificity						
pLDH	293	58.0	52.1–63.8	1252	92.6	91.1–94.0
HRP2	293	57.4	51.5–63.1	1252	93.5	91.9–94.8
PPV						
pLDH	361	66.3	61.1–71.2	233	60.5	53.9–66.8
HRP2	370	66.3	61.2–71.1	215	61.9	55.0–68.4
NPV						
pLDH	169	98.8	95.8–99.9	1173	98.8	98.0–99.3
HRP2	169	98.8	95.8–99.9	1192	98.2	97.2–98.8

*PPV* positive predictive value, *NPV* negative predictive value

^a^For sensitivity, N represents all microscopy-positives; for specificity, N represents all microscopy-negatives; for PPV, N represents all test positives; and for NPV, N represents all test negatives

^‡^Exact McNemar test p = 0.008

During the high season, the sensitivity of both tests was 100% at all except the lowest levels of parasitaemia (< 200 parasites/µl); during the low season, the sensitivities at the lowest level of parasitaemia were similar to those seen during the high season, but inferior in the intermediate levels of parasitaemia (Table 4). Nonetheless, it should be noted that the confidence intervals around the low-season estimations are much wider, as there were fewer positive results, particularly at higher levels of parasitaemia.

**Table 4:** Sensitivity of two malaria rapid diagnostic tests by parasite density, Magaria, Niger, 2017–2018

Test	High season			Low season		
Parasites/µl	N with given parasitaemia	Value	95% CI	N with given parasitaemia	Value	95% CI
pLDH	246	99.2	97.0–99.9	155	91.0	85.3–95.0
< 200	11	81.8	48.2–97.7	54	81.5	68.6–90.7
200–1999	42	100	91.4–100	54	94.4	84.6–98.8
2000–199,999	179	100	97.9–100	46	97.8	88.5–99.9
≥ 200,000	14	100	76.8–100	1	100	2.5–100
HRP2	246	99.2	97.1–99.9	155	85.8	79.3–90.9
< 200	11	81.8	48.2–97.7	54	75.9	62.4–86.5
200–1999	42	100	91.6–100	54	85.2	72.9–93.4
2000–199,999	179	100	98.0–100	46	97.8	88.5–99.9
≥ 200,000	14	100	76.8–100	1	100	2.5–100

### Post-hoc analyses

A series of exploratory analyses not originally planned was undertaken to explain the modest performance of the pLDH test. As Table 5 shows, there were relatively few discrepancies in the results obtained by the two RDTs during both seasons (0.9% of participants during the low season and 1.3% during the high season). Of the participants with valid discordant results between the two tests, in the high season, all 3 were negative by microscopy. In the low season, all 18 discordant results were positive by pLDH and negative by the HRP2 test. Seven of the 18 were negative by microscopy, and the median parasitaemia for the remaining 11 was 570 parasites/µl (IQR 199–1684, range 40–3244). For both tests, performance was similar across different production lots (Table 6).

**Table 5:** Results of HRP2 and pLDH rapid diagnostic tests for individual samples, Magaria, Niger, 2017–2018

High season			Low season		
HRP2			HRP2		
pLDH	Positive	Negative	pLDH	Positive	Negative
Positive	360	1	Positive	215	18
Negative	2	167	Negative	0	1173
Invalid	8	1	Invalid	0	1

**Table 6:** Performance characteristics of the two malaria rapid diagnostic tests by production lot, Magaria, Niger, 2017–2018

	Test	High season			Low season		
	Lot number	N^a^	Value	95% CI	N^a^	Value	95% CI
Sensitivity	pLDH	246	99.2	97.0–99.9	155	91.0	85.3–95.0
	MN16C61	129	99.2	95.7–100	56	87.5	75.9–94.8
	MN16E61	117	99.1	95.2–100	48	89.6	77.3–96.5
	MN16G62	–	–	–	51	96.1	86.5–99.5
	HRP2	246	99.2	97.1–99.9	155	85.8	79.3–90.9
	05CDB181A	226	99.1	96.8–99.9	–	–	–
	05CDB101A	–	–	–	69	84.1	73.3–91.8
	05CDB232A	–	–	–	86	87.2	78.3–93.4
Specificity	pLDH	293	58.0	52.1–63.8	1252	92.6	91.1–94.0
	MN16C61	131	57.4	48.4–66.0	353	94.6	91.7–96.7
	MN16E61	162	58.5	50.4–66.2	442	91.4	88.4–93.8
	MN16G62	–	–	–	457	92.3	89.5–94.6
	HRP2	293	57.4	51.5–63.1	1252	93.5	91.9–94.8
	05CDB181A	269	56.2	50.0–62.2	–	–	–
	05CDB101A	–	–	–	487	93.0	90.4–95.1
	05CDB232A	–	–	–	765	93.7	91.8–95.3

Production lots 05CDB078A (24 participants) and 05FDC006A (20 participants) of the HRP2 RDT were also used during the high season, but given the small number of tests, lot-specific characteristics are not reported here. This explains the slight differences between the overall and lot-specific performance of the HRP2 tests in the high season

^a^For sensitivity, N represents all true positives; for specificity, N represents all true negatives tested with a given production lot

There were no differences in performance characteristics of the test by participant gender. Performance characteristics were broadly similar by age in both transmission seasons, although the number of participants in each age group was small and the study was not designed to evaluate this (Table 7). Importantly, during the high transmission season, there were no differences in performance characteristics among children who did/did not receive the most recent distribution of SMC. The performance characteristics of the tests did not differ between different weeks of the SMC distribution cycle (Table 8), when the protective effect of the intervention might have differed slightly due to changing drug levels over time. When children with gametocytaemia (in the absence of asexual forms) were also considered to be microscopy-positive, overall performance also remained similar (Additional file 1: Table S1).

**Table 7:** Performance characteristics of two malaria rapid diagnostic tests by participant age, Magaria, Niger, 2017–2018

	Test	High season			Low Season		
	Age in months	N^a^	Value	95% CI	N^a^	Value	95% CI
Sensitivity	pLDH	246	99.2	97.0–99.9	155	91.0	85.3–95.0
	3–11	45	100	92.1–100	34	91.2	76.3–98.1
	12–23	58	100	93.7–100	53	86.8	74.7–94.5
	24–35	79	97.4	90.8–99.7	39	92.3	79.1–98.4
	≥ 36	64	100	94.2–100	29	96.6	82.2–99.9
	HRP2	246	99.2	97.1–99.9	155	85.8	79.3–90.9
	3–11	45	100	92.1–100	34	85.3	68.9–95.0
	12–23	58	100	93.8–100	53	81.1	68.0–90.6
	24–35	79	97.5	91.2–99.7	39	89.7	75.8–97.1
	≥ 36	64	100	94.4–100	29	89.7	72.6–97.8
Specificity	pLDH	293	58.0	52.1–63.8	1252	92.6	91.1–94.0
	3–11	103	73.3	63.5–81.6	523	96.2	94.2–97.6
	12–23	108	53.3	43.4–63.0	473	92.0	89.1–94.3
	24–35	50	41.7	27.6–56.8	166	87.9	81.9–92.4
	≥ 36	32	50.0	31.9–68.1	90	84.4	75.3–91.2
	HRP2	293	57.4	51.5–63.1	1252	93.5	91.9–94.8
	3–11	103	73.8	64.2–82.0	523	96.6	94.6–97.9
	12–23	108	52.3	42.5–62.1	473	93.0	90.3–95.1
	24–35	50	38.8	25.2–53.8	166	89.2	83.4–93.4
	≥ 36	32	50.0	31.9–68.1	90	85.6	76.6–92.1

^a^For sensitivity, N represents all true positives; for specificity, N represents all true negatives tested with a given test lot

**Table 8:** Performance characteristics of two malaria rapid diagnostic tests by week since most recent seasonal malaria chemoprevention distribution, Magaria, Niger, 2017

	Test	High season		
	Week since SMC	N	Value	95% CI
Sensitivity	pLDH	246	99.2	97.0–99.9
	1	55	100	94.5–100
	2	58	100	93.5–100
	3	68	100	93.4–100
	4	65	97.0	89.5–99.6
	HRP2	246	99.2	97.1–99.9
	1	55	100	94.5–100
	2	58	100	93.5–100
	3	68	100	93.8–100
	4	65	97.1	89.8–99.6
Specificity	pLDH	293	58.0	52.1–63.8
	1	134	57.9	49.0–66.4
	2	45	66.7	51.0–80.0
	3	47	52.3	36.7–67.5
	4	67	56.1	43.3–68.3
	HRP2	293	57.4	51.5–63.1
	1	134	57.1	48.3–65.7
	2	45	66.7	51.0–81.0
	3	47	52.2	36.9–67.1
	4	67	55.2	42.6–67.4

Regarding external quality control of microscopy, during the high transmission season, among 25 negative slides, there was 100% concordance with the reference laboratory. Among 30 positive slides, 29 were read to have the same species (*P. falciparum*), and 1 which was interpreted as *P. ovale* was read as *P. falciparum* by the external laboratory. During the high season, the overall difference in parasite densities between the laboratories was 8%, and there were no differences in the classification of parasite density into the categories shown in Table 2. During the low transmission season, among 30 negative slides, 28 were read as being negative by the reference laboratory, and 2 were read as *P. falciparum*, both with a parasite density of 40 parasites/µl. Among 17 positive slides, there was only one discordance: a sample with a parasite density of 24 parasites/µl in the study laboratory was read as negative in the reference laboratory. Otherwise there was no difference in the classification of parasite density into the categories shown in Table 2.

Visual inspection of all photographs was performed, with attention to the photographs of RDT-positive/microscopy-negative pLDH tests and positive RDTs with low parasitaemia on microscopy. The lines given by pLDH test (control and test lines) were generally fainter and slightly less crisp than the lines (control and test) given by the HRP2 test. This difference was noticeable when looking at the tests side by side, as illustrated in Additional file 2, which shows the photograph of the tests of a high-season participant who had a negative blood smear and two positive RDTs.

Among the participants during the high season with positive RDTs but negative microscopy results, a majority had test bands of equal intensity to the control band (Additional file 2), but approximately one-quarter had test bands that were clearly visible but markedly less strong than the control bands on both tests (Additional file 3). During the low season, the patterns were inversed, with most patients with RDT-positive/microscopy-negative results having test bands markedly less strong than the control band (as in Additional file 4), and approximately one-quarter with test bands of equal intensity.

During the high season, 7 of the 8 participants who had parasite densities ≤ 200/µl had strong bands for both the pLDH and HRP2 tests. In the low season, when RDTs were positive at the lowest parasite densities (≤ 50 sexual forms/µl), the test bands were generally weak on both tests, as exemplified by Additional file 5.

## Discussion

The specificity and PPV of the pLDH RDT were low, much lower than previously described, and very similar to the HRP2 test, which was unexpected considering the different performance and characteristics of these types of tests reported previously [1, 3–5, 9]. This result is both difficult to explain and potentially problematic as the pLDH test is scaled up across different high-transmission environments.

The prevalence of parasitaemia during the high transmission season was slightly lower than expected (46% vs the 60% expected among febrile children presenting to a health centre), which may be due to the protective effect of SMC. Previous evaluations of the pLDH and HRP2 tests have not been performed in areas receiving SMC. Nonetheless, it is unclear how SMC would affect the performance of an RDT. One possibility would be that SMC could lead to high levels of sub-microscopic parasitaemia, some of which would be detected by the rapid tests but not by microscopy, a phenomenon described elsewhere outside the setting of SMC [10]. This could potentially explain why false positive results during the high season were identical with the HRP2 and pLDH tests. However, it could be expected that the proportion of sub-microscopic infection would change over the course of an SMC distribution, as the protective effect of the drug combination wanes. Thus, the fact that the characteristics remained similar over the course of an SMC distribution cycle would weaken this hypothesis. Although the specificity of the pLDH test improved during the low season, the positive predictive value was still poor. Importantly, the specificity did not vary by production lot.

The second surprising result of this study is the poor sensitivity of the pLDH (91.0%, 95% CI 85.3–95.0) and particularly HRP2 (85.8%, 95% CI 79.3–90.9) tests during the low malaria transmission season. These sensitivities are lower than would be expected [3–5], even given the overall lower parasite burden during the low transmission season. Indeed, even when restricting the analysis to specimens with parasitaemia > 200 parasites/µl, sensitivities were still low, particularly for the HRP2 test, given that rapid tests based on this antigen typically have sensitivities which approach 100% at these levels of parasitaemia [3, 11, 12]. Even more surprising is that the sensitivity of the HRP2 test was lower than that of the pLDH test, which goes against previous experience that HRP2 tests are more sensitive than pLDH tests [3, 12].

The photographs of RDT cassettes provide a valuable record of what tests look like when performed on ‘real-life’ samples, and are an added value for monitoring, especially given the surprising results obtained. The intensity of bands should not be correlated with severity of infection, but nonetheless, in this evaluation, interesting patterns emerged. The visual appearance of the pLDH test is different from the HRP2 test, with a fainter, slightly hazier line than the HRP2 test. While this does not call into question the accuracy of the test, the experience in the field was that this was less desirable for staff accustomed to the clearer lines of the HRP2 test.

Another hypothesis for the discrepant results between the two tests in the low season would be the presence of HRP2 deletions in the parasite genome, which in turn cause HRP2-based RDTs to be negative even in the presence of *Plasmodium* [13]. This has been recognized as a problem in some parts of Africa, including Kenya and Eritrea [14, 15]. A nationwide survey in the Democratic Republic of Congo suggested that 6.4% of infections had had HRP2 deletions, and that there was significant spatial clustering [16]. The absence of such discrepancies (pLDH+/HRP2−) in the high transmission season in Magaria suggests that the HRP2 deletions are probably not widespread in the area, but it should be monitored.

One of the limitations of this study is that information about recent use of artemisinin combination therapy (ACT) was not collected. One hypothesis to explain the high false-positivity rate would be that persistent antigenaemia remained high after effective ACT treatment, but given the characteristics of the time to become negative of these tests, all of these false positives would have had to present to the clinic very quickly after finishing their ACT, which seems improbable. The RDTs were stored at ambient temperatures for relatively short periods of time, and those temperatures may have surpassed recommended storage temperatures, so degradation might have been of concern. On the other hand, this mimics usual storage condition for the vast majority of RDTs used in routine clinical care in the Sahel.

PCR was not performed on these samples, which may be a limitation of this study. PCR comes with certain advantages, including that it relies on a standardized process, with less room for human error in interpretation of a result than microscopy, and also that it has a higher ability to detect low-level parasitaemia [17, 18]. For these reasons, PCR and ultra-sensitive RDTs have become standard in malaria elimination settings [19]. On the other hand, in a setting such as Niger during the high transmission season, one could question the clinical significance of a PCR assay that detected a very low-level sub-microscopic parasitaemia that would less likely be the cause of a febrile child’s acute illness [20, 21]. Therefore, in this setting, where the goal was to differentiate clinical malaria from non-malaria, PCR was not chosen as a comparator, relying instead on microscopy as a gold standard, which was believed to be a better indicator of clinical illness in this setting. Microscopy, like PCR and other diagnostic tools, is operator-dependent, but given the results here, the usual lower limits of detection for expert microscopists (between 50 and 100 parasites/µl) seem to have been applicable in this case. Theoretically, it is possible that another antigen-detection method such as ELISA would have been a more appropriate comparator for antigen-based RDTs given that correlations between antigen concentration and parasite level is different for HRP2 and pLDH [22, 23]. However, malaria antigen-specific ELISAs are not routinely available and the study site was not equipped for sample storage for this purpose. More importantly, using ELISA would not have met with the objective of assessing the clinical performance of the RDTs in routine conditions.

## Conclusions

In summary, this clinical diagnostic evaluation was undertaken to support the routine use of the pLDH RDTs, but these results showed that the tests had similar performance to an HRP2 test, particularly regarding specificity during the high transmission season. This likely led to a significant amount of over-treatment of malaria, though in this setting, over-treatment is preferable to under-treatment. Nonetheless, as the pLDH test continues to be introduced into new settings, many questions remain. It should be monitored in areas with and without SMC, and future evaluations should include additional PCR analyses, possibly including consideration of HRP2 deletions.

## Supplementary information

**Additional file 1: Table S1.** Performance characteristics of two malaria rapid diagnostic tests when considering children with asexual forms as well as those with gametocytemia (in the absence of asexual forms) as being parasitemic, Magaria, Niger, 2017–2018^*^.

**Additional file 2.** Photograph of HRP2 (blue text) and pLDH (black text) malaria RDTs, high transmission seasons, Magaria, Niger. This participant had a negative blood smear.

**Additional file 3.** Photograph of HRP2 (blue text) and pLDH (black text) malaria RDTs, high transmission season, Magaria, Niger. This participant had a negative blood smear.

**Additional file 4.** Photograph of HRP2 (blue text) and pLDH (black text) malaria RDTs, low transmission season, Magaria, Niger. This participant had a negative blood smear.

**Additional file 5.** Photograph of HRP2 (blue text) and pLDH (black text) malaria RDTs, low transmission season, Magaria, Niger. This participant had 24 parasites/mcl on blood smear.

## Data Availability

The datasets generated and analyzed during this study are available from the corresponding author on reasonable request.

## Abbreviations

ACT: artemisinin-based combination therapy
CSI: integrated health center (*Centre de Santé Intégré*)
HRP2: histidine-rich protein 2
NPV: negative predictive value
PDS: panel detection score
pLDH: *Plasmodium* lactate dehydrogenase
PPV: positive predictive value
RDT: rapid diagnostic test
SMC: seasonal malaria chemoprevention
WHO: World Health Organization
