# Supplementary material for: Clinical diagnostic evaluation of HRP2 and pLDH-based rapid diagnostic tests for malaria in an area receiving seasonal malaria chemoprevention in Niger
Source: Malar J. 2019 Dec 26;18:443. doi: 10.1186/s12936-019-3079-1 (PMC6933886; doi:10.1186/s12936-019-3079-1)
Supplement: Supplementary file 1 — Additional file 1: Table S1. Performance characteristics of two malaria rapid diagnostic tests when considering children with asexual forms as well as those with gametocytemia (in the absence of asexual forms) as being parasitemic, Magaria, Niger, 2017–2018*. [file 12936_2019_3079_MOESM1_ESM.docx]

Additional Table S1: Performance characteristics of two malaria rapid diagnostic tests when considering children with asexual forms as well as those with gametocytemia (in the absence of asexual forms) as being parasitemic, Magaria, Niger, 2017-2018^*^

|  | **High transmission season** | | |  | **Low transmission season** | | |
| --- | --- | --- | --- | --- | --- | --- | --- |
| **Characteristic** Test | N^†^ | Value | 95%CI |  | N^†^ | Value | 95%CI |
| **Sensitivity** |  |  |  |  |  |  |  |
| pLDH | 264 | 98.1 | 95.5-99.4 |  | 178 | 86.0 | 80.0-90.7 |
| HRP2 | 264 | 98.1 | 95.6-99.4 |  | 178 | 80.9 | 74.3-95.5 |
|  |  |  |  |  |  |  |  |
| **Specificity** |  |  |  |  |  |  |  |
| pLDH | 275 | 60.3 | 54.2-66.2 |  | 1229 | 93.5 | 92.0-94.8 |
| HRP2 | 275 | 59.6 | 53.6-65.5 |  | 1229 | 94.2 | 92.8-95.5 |
|  |  |  |  |  |  |  |  |
| **PPV** |  |  |  |  |  |  |  |
| pLDH | 361 | 70.1 | 65.1-74.8 |  | 233 | 65.7 | 59.2-71.7 |
| HRP2 | 370 | 70.0 | 65.0-74.6 |  | 215 | 67.0 | 60.3-73.2 |
|  |  |  |  |  |  |  |  |
| **NPV** |  |  |  |  |  |  |  |
| pLDH | 169 | 97.0 | 93.2-99.0 |  | 1173 | 97.9 | 96.9-98.6 |
| HRP2 | 169 | 97.0 | 93.2-99.0 |  | 1192 | 97.1 | 96.0-98.0 |
|  |  |  |  |  |  |  |  |

^*^ PPV: positive predictive value; NPV: negative predictive value

^†^ For sensitivity, N represents all microscopy-positives; for specificity, N represents all microscopy-negatives; for PPV, N represents all test positives; and for NPV, N represents all test negatives
