# Supplementary material for: Clinical diagnostic evaluation of HRP2 and pLDH-based rapid diagnostic tests for malaria in an area receiving seasonal malaria chemoprevention in Niger
Source: Malar J. 2019 Dec 26;18:443. doi: 10.1186/s12936-019-3079-1 (PMC6933886; doi:10.1186/s12936-019-3079-1)
Supplement: Supplementary file 4 — Additional file 4. Photograph of HRP2 (blue text) and pLDH (black text) malaria RDTs, low transmission season, Magaria, Niger. This participant had a negative blood smear. [file 12936_2019_3079_MOESM4_ESM.docx]

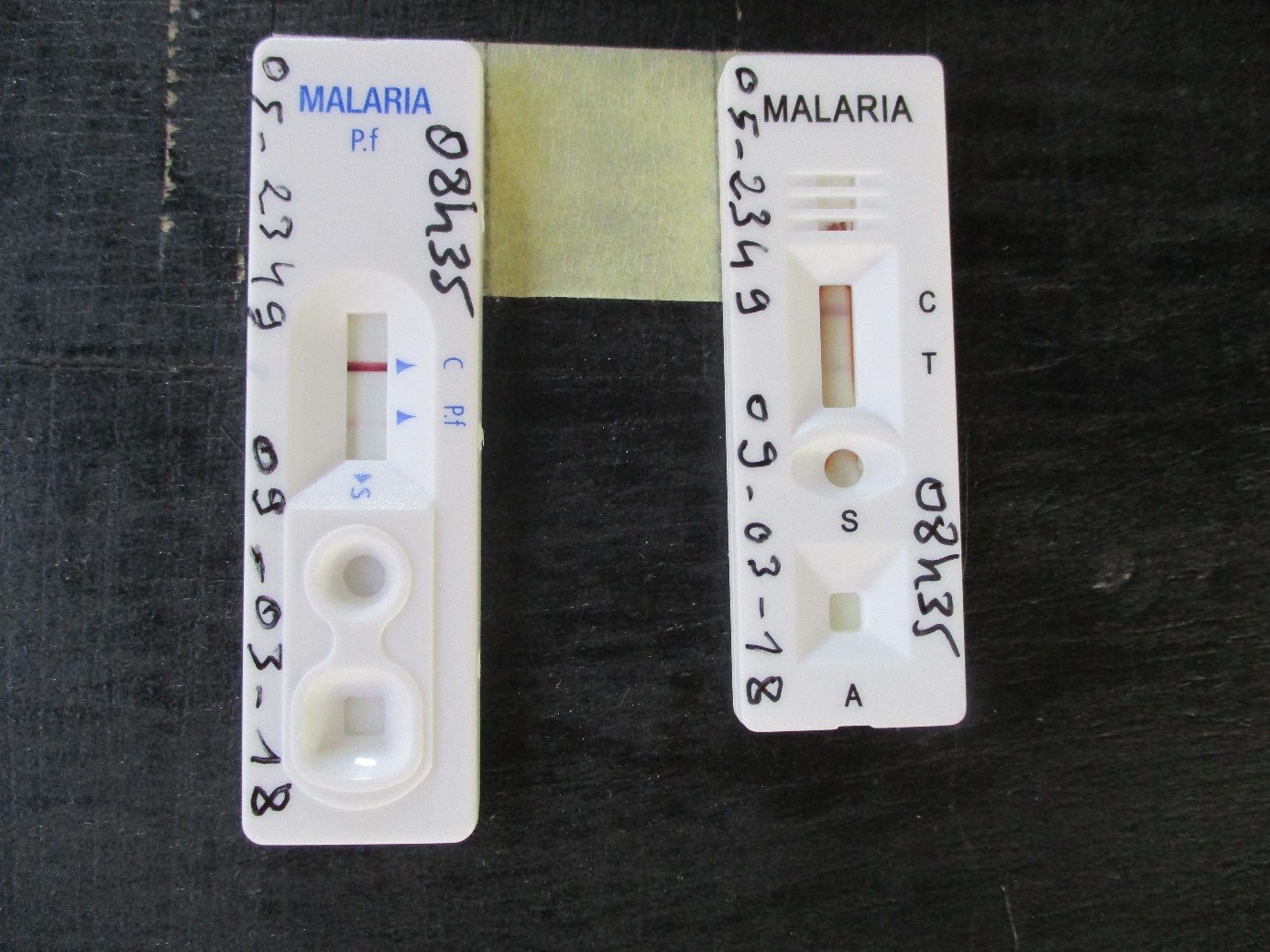


Additional file 4: Photograph of HRP2 (blue text) and pLDH (black text) malaria RDTs, low transmission season, Magaria, Niger. This participant had a negative blood smear.
